# Ultrafast Excited-State Dynamics of the Organic Photoredox Catalyst DDQ

**DOI:** 10.1021/acs.jpclett.5c02391

**Published:** 2025-09-09

**Authors:** Deborin Ghosh, Vera Brieskorn, Charlotte A. Smith, Hallam J. M. Greene, Ria G. Binyahan, Federico J. Hernández, Alastair J. J. Lennox, Basile F. E. Curchod, Andrew J. Orr-Ewing

**Affiliations:** School of Chemistry, 1980 University of Bristol, Cantock’s Close, Bristol BS8 1TS, U.K.

## Abstract

The electron-deficient oxidant 2,3-dichloro-5,6-dicyano-1,4-benzoquinone (DDQ) has recently emerged as a promising visible-light photoredox catalyst. However, its excited-state behavior remains poorly understood. Here, we investigate the ultrafast dynamics of photoexcited DDQ in acetonitrile using transient electronic and infrared absorption spectroscopy, supported by quantum chemical calculations. Upon 395 nm excitation, we identify rapid intersystem crossing (ISC) from the singlet to triplet manifold within 1.5 ps, followed by internal conversion and vibrational relaxation on a 10.9 ps time scale. Our findings demonstrate that DDQ exhibits near-unity ISC quantum yield and long triplet lifetime. Together with the high reduction potential of the triplet state, these properties make it a viable metal-free alternative to conventional iridium- and ruthenium-based photocatalysts.

## Graphical abstract

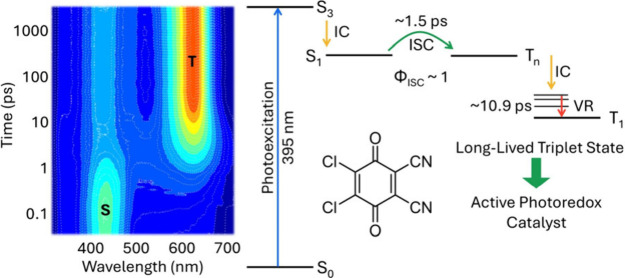

Activation of chemically inert C–H bonds, which possess high bond dissociation energies (∼410 kJ/mol), is highly advantageous in organic synthesis, especially in the pharmaceutical and related industries.^1–4^ Efforts have been ongoing for several decades, with ruthenium (Ru) and iridium (Ir) metal complexes emerging as the most efficient and widely used photocatalysts for these activation reactions.^5–7^ Due to the low abundance and high cost of these two transition metals, significant endeavor is focused on developing more cost-effective and efficient alternatives. Recently, several attempts have been reported to replace Ru and Ir complexes with 3d transition-metal complexes.^8,9^ Additionally, efforts have also been made to employ metal-free, visible-light photoredox catalysts using organic dyes. Some common organic photoredox catalysts include cyanoarenes, benzophenones, quinones, xanthenes and thiazines.^10–13^ Recent studies indicate that another quinone derivative, 2,3-dichloro-5,6-dicyano-1,4-benzoquinone (DDQ, with the structure shown in Figure 1(a)), also has the potential to replace expensive transition metal complexes as a photoredox catalyst.^14,15^ Importantly, DDQ is inexpensive, readily available, and already widely used as an oxidant (reduction potential 0.51 V vs standard calomel electrode (SCE)^16^) in organic synthesis.^17^ The oxidation power of DDQ is reported to increase significantly upon photoexcitation,^14^ reaching 3.18 V (vs SCE) in its triplet state, thereby enabling challenging synthetic transformations such as C–H bond activation. Functionalization of arene, alkene C­(sp^2^)–H, or alkyl C­(sp^3^)–H bonds typically proceeds via two-electron processes; however, there are only a few reports of efforts to understand the mechanistic pathways mediated by photoexcited DDQ,^14,18–21^ and further exploration is still needed. Moreover, the ultrafast excited-state dynamics of DDQ following photoexcitation have not been comprehensively explored.

**Figure 1:**
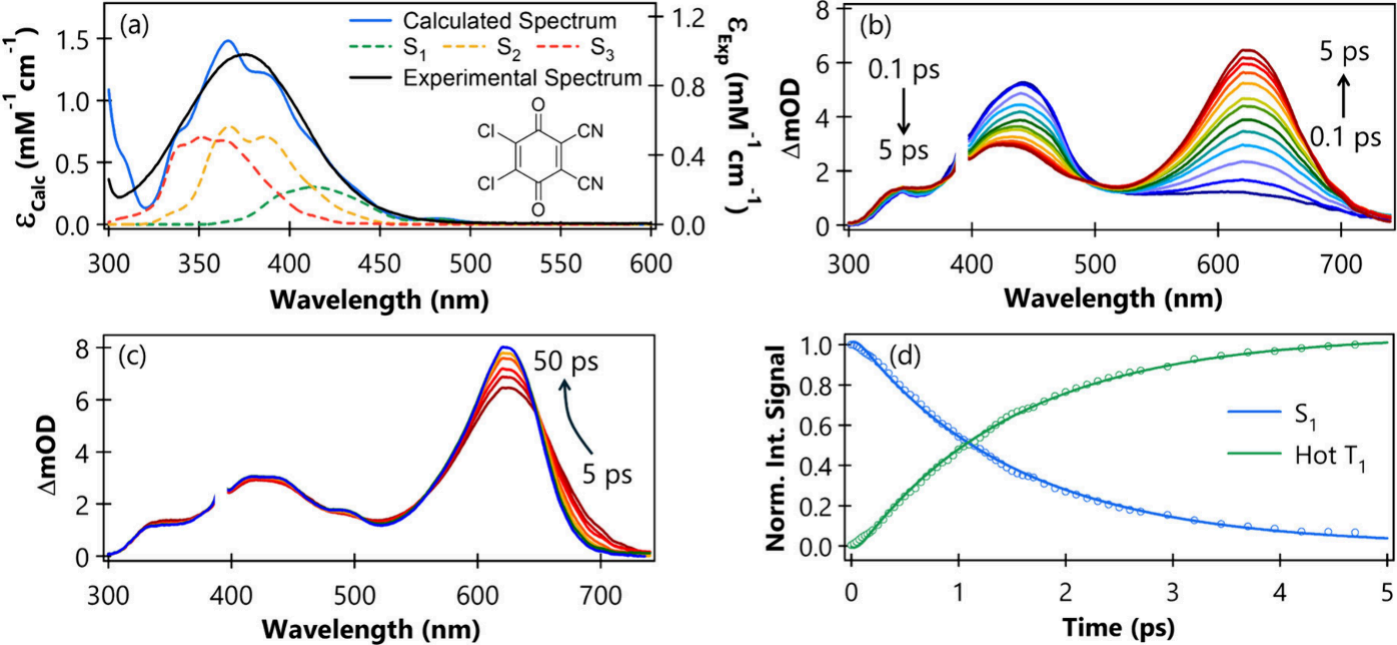
(a) UV–Vis absorption spectrum (black solid line) of 30 mM DDQ in MeCN, the calculated absorption spectrum (blue solid line, shifted by −0.24 eV), and the individual contributions from the S_1_, S_2_, and S_3_ states (dashed lines). Details of the calculation methods are given in Section S1.2 in the SI. Note the separate extinction coefficient (ε) scales for calculated and experimental spectra. The molecular structure of DDQ is shown as an inset. (b) TA spectra of 30 mM DDQ in MeCN following 395 nm excitation, recorded at early time delays (100 fs to 5 ps). (c) TA spectra at later time delays (5 to 50 ps). (d) Kinetics of S_1_ state population decay and hot T_1_ state growth up to 5 ps, derived from time-dependent integrated and normalized band intensities following spectral decomposition using KOALA software.^25^ Solid lines represent instrument response function (IRF) convoluted exponential fits.

In this study, we present a detailed experimental investigation of the ultrafast photophysical events that occur prior to the formation of the long-lived triplet state, supported by quantum chemical calculations to map the relevant excited-state energy landscape. Separately, we have also examined the photoredox reactivity of DDQ in the presence of various benzylic C–H containing substrates; the mechanistic insights will be discussed in detail in a separate publication.

The electronic absorption spectrum of DDQ in acetonitrile (MeCN), as shown in Figure 1(a), reveals that although the absorption maximum is around 375 nm, the molecule retains significant absorptivity in the visible range (λ ≥ 400 nm), making it suitable for use as a visible-light photocatalyst. A few absorption spectra reported in the literature^15,22^ differ from those presented in this study, likely due to variations in experimental conditions or sample preparation. Although the absorption spectrum of DDQ in MeCN obtained in our study closely resembles that reported by Berto and co-workers,^23^ and the position of the absorption maximum aligns well with the data reported by Miller and co-workers,^24^ we observed a noteworthy phenomenon during sample handling. Specifically, the yellow color of the DDQ solution in MeCN turned red upon contact with metal surfaces of the Harrick cell used for transient absorption spectroscopy, suggesting the formation of reduced DDQ species via electron transfer from the metal. To prevent such contamination, we strictly avoided the use of metal-containing laboratory tools such as spatulas and needles during the handling of DDQ and its solutions. The purity of the DDQ solutions used in our experiments was further verified by ^13^C NMR spectroscopy (shown in Figure S1 in the Supporting Information (SI)). Quantum-chemistry calculations reported here show that the high absorbance of DDQ at around 370 nm is caused by a transition from the ground electronic state to an electronic state with ππ* character, corresponding to S_3_ at the Franck–Condon (FC) point (ground-state equilibrium geometry). Two other singlet excited electronic states can be found for λ ≥ 300 nm, S_1_ and S_2_, with *n*_–_π* and *n*_*+*_π* character, respectively. Here, the *n* symbol indicates a combination of the carbonyl lone pairs, either in phase (*n*_+_) or out of phase (*n*_–_). Non-Condon effects lead to character mixing (and intensity borrowing) between the three lowest excited electronic states, as shown in the computed absorption spectrum in Figure 1(a)). Computational details are provided in Section S1 and Figures S2–S6 of the SI.

We employed transient electronic absorption (TA) and time-resolved infrared (TRIR) spectroscopy to monitor changes in the electronic and vibrational energy levels, respectively, over time delays ranging from 100 fs to 3.75 ns following photoexcitation of DDQ in MeCN using a 395 nm pump wavelength. Although the absorption maximum of DDQ is at 375 nm, 395 nm excitation was selected to match the wavelength of LEDs commonly used in organic synthesis. The transient spectroscopy setup has been described in detail in our previous publication;^26^ a brief overview is provided in Section S2 in the SI. As previously noted, all direct contact between DDQ solutions and metal surfaces was avoided. Consequently, a flow cell could not be used during TA measurements to refresh the sample. Instead, to minimize photobleaching, a static sample was continuously translated in both X and Y directions on an XY stage, perpendicular to the pump–probe plane, throughout the measurement. No signs of photodegradation were observed upon exposure to ∼ 200 nJ, 395 nm pump pulses with a spot size of 250–300 μm.

TA spectra of 30 mM DDQ in MeCN, recorded from 100 fs to 5 ps, are presented in Figure 1(b). At the earliest time delays, an excited-state absorption (ESA) band centered at 433 nm, with a weaker shoulder extending up to 700 nm, is observed. This band rapidly evolves within 5 ps into a dual ESA feature centered at 425 and 625 nm. Both bands become progressively sharper up to 50 ps as shown in Figure 1(c), although their peak positions remain unchanged. As the spectral features sharpen, a minor band near 496 nm becomes clearly distinguishable after ∼10 ps. We also observe slight changes in a weak TA feature located near 350 nm, as shown in Figures 1(b) and 1(c). Because this feature overlaps ground-state bleach features in the TA spectra, and it lies at the edge of the white-light continuum used in the TA experiment, we do not consider it further. No significant changes in intensity or spectral position are observed for time delays longer than 10 ps; the transient signals remain unchanged up to 3.75 ns, as shown in Figure S7 in the SI. This latter time delay corresponds to the upper limit of the optical delay line in our TA setup at the University of Bristol. The 433 nm band and its associated long-wavelength shoulder are assigned to the S_1_ ESA, supported by calculations discussed below.

Quantum-chemical calculations provide insights into the photophysical deactivation of DDQ. At its equilibrium geometry (FC point), DDQ is photoexcited to the S_3_(ππ*) electronic state (see NTOs in the lower panel of Figure 2) and can relax toward S_1_ *diabatically*, i.e., by preserving its ππ* character, as depicted by the barrierless interpolated pathway between the ground-state geometry, FC, and the minimum-energy geometry of the S_1_(ππ*) state in Figure 2. The nonemissive nature of DDQ from this S_1_(ππ*) state, as determined from steady-state fluorescence measurements, suggests that the excited singlet state is short-lived. Therefore, the long-lived dual absorption bands at 425 and 625 nm cannot be attributed to singlet-state ESA. In previous reports,^14,19,20,27^ the transient band near 625–630 nm was assigned to triplet ESA; however, this assignment was primarily based on the band’s long-lived nature and comparisons with other substituted quinones. To date, no direct quenching studies using established triplet quenchers have been reported to support this assignment. The possibility of radical cation formation via photoinduced electron ejection to the solvent has also not been considered, although it appears unlikely given DDQ’s strongly electron-deficient character.^14,16^ Quantum chemical calculations indicate that DDQ can undergo efficient intersystem crossing to low-lying triplet states (dashed curves in Figure 2) upon reaching the region of the S_1_(ππ*) minimum. More specifically, the spin–orbit coupling magnitude between the ^1^ππ* and ^3^ *n*_–_π* states is the only sizable one, with a value of 29.9 cm^–1^ (LR-TDDFT/TDA/ωB97X-D4/def2-TZVP/PCM). An intersystem crossing rate calculation, incorporating the effect of vibrational motion, predicts a fast ISC process between these two electronic states with a time constant of 11 ps. Details of the methods used to calculate the ISC transition rate are given in Section S1.5 in the SI. The normal mode dominantly responsible for this fast ISC process is depicted as an inset in Figure 2 and shows a molecular distortion occurring in the plane of DDQ with calculated wavenumber *v̅* = 367 cm^–1^ (see Figure S6 in the SI for further details).

Static calculations of excited-state transitions (SCS-CC2/def2-TZVP/COSMO) from the S_1_(ππ*) minimum and the T_1_(ππ*) minimum give, in both cases, two bright vertical transitions in the range probed experimentally: 340 nm (with oscillator strength f = 0.1628) from S_1_(ππ*) and 357 nm (f = 0.1493) from T_1_(ππ*), and 488 nm (f = 0.1086) from S_1_(ππ*) and 523 nm (f = 0.0986) from T_1_(ππ*). Based on these quantum chemical insights, the dual bands at 425 and 625 nm can be assigned to T_1_ ESA, emerging as a similar, but slightly shifted dual ESA feature as the S_1_ ESA disappears. Overall, these findings explain the quick decay of the S_1_ ESA bands and the subsequent appearance of the long-lived dual ESA bands at 425 and 625 nm, which are thus assigned to triplet ESA. The progressive sharpening of the triplet ESA bands over time could arise from internal conversion (IC) and vibrational relaxation (VR) from the ^3^ *n*_–_π* state to the T_1_ minimum with ^3^ππ* character (see interpolated pathway in Figure 2). However, because no positional shifts of these bands were observed, we conclude that IC from the ^3^ *n*_–_π* state to the T_1_(ππ*) state is not detected experimentally. Instead, the sharpening of the T_1_ ESA bands is attributed to VR from the hot T_1_ state to its minimum.

**Figure 2:**
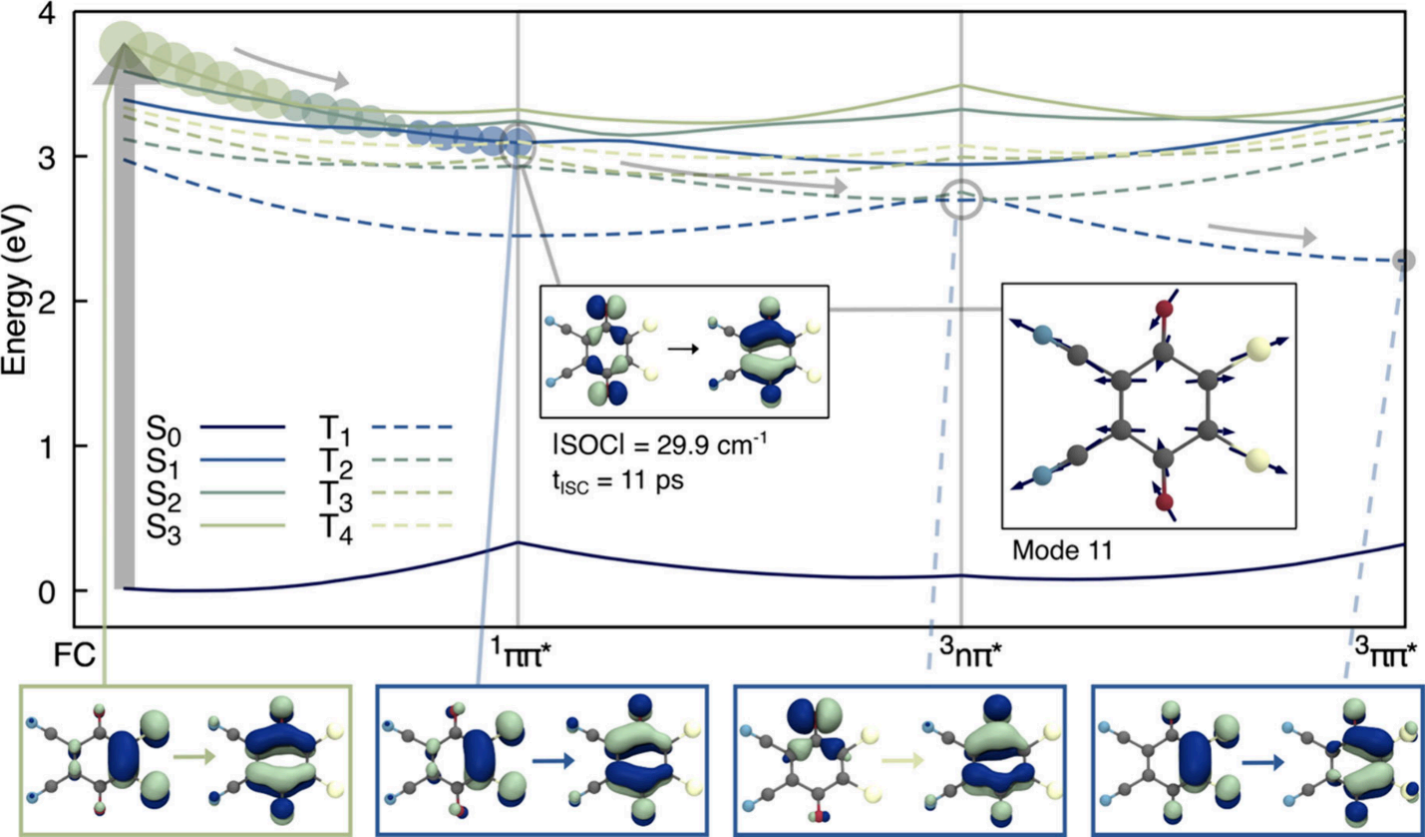
Deactivation pathways for photoexcited DDQ along interpolated paths. Critical points on DDQ potential energy surfaces—minima for S_0_ (FC), S_1_ (^1^ππ*), T_1_ (^3^n_–_π*), and T_1_ (^3^ππ*)—were obtained with (LR-TD)­DFT/TDA/ωB97X-D4/def2-TZVP/PCM­(MeCN) and connected by geodesic paths. Electronic energies and oscillator strengths (whose magnitudes in the singlet manifold are indicated by circles of proportional radii) were recalculated along these paths at the SCS-CC2/def2-TZVP/COSMO­(MeCN) level of theory for singlet (plain curves) and triplet (dashed curves) electronic states. Arrows indicate the suggested deactivation along a pathway initiated in a state with dominant ^1^ππ* character to T_1_(^3^ππ*), with an inset for the intersystem crossing process and the natural transition orbitals (NTOs) characterizing the ^3^n_–_π* electronic character (at the ^1^ππ* optimized geometry). |SOC| designates the SOC magnitude between the electronic states of ^1^ππ* and ^3^n_–_π* character. The right inset depicts the vibrational mode (mode 11, *v̅* = 367 cm^–1^) dominantly participating in the intersystem crossing process. The bottom inset displays the NTOs for each critical structure, highlighted by their corresponding color code.

To extract kinetic information from the experimental measurements, we performed spectral decomposition of the TA data using the KOALA program.^25^ The TA spectrum at 100 fs was selected as the basis function for the S_1_ ESA, as it represents the earliest clean transient absorption signal. Signals recorded from 0–100 fs were excluded because of contamination by stimulated Raman scattering from the solvent. The 5 ps TA spectrum was chosen as the basis for the hot T_1_ ESA, as this corresponds to a time point at which the decay of the S_1_ ESA band is complete, but vibrational cooling is still occurring. Beyond 5 ps, the positions of the TA bands remain unchanged; however, a pronounced spectral narrowing is observed up to 50 ps due to VR from the hot T_1_ state to its minimum. Since no further spectral evolution occurs beyond 50 ps, the TA spectrum at this time delay was used as the basis spectrum for the vibrationally relaxed T_1_ ESA. All TA spectra were well fitted using these basis spectra, as demonstrated in Figures S8­(a) and S8­(b) in the SI, which show representative decompositions at 1.25 and 13 ps, respectively. The integrated areas of the fitted basis functions represent the band intensities at each time delay, and the temporal evolution of these intensities reflects the kinetics of the corresponding transient species. The extracted kinetic traces for the S_1_ ESA, hot T_1_ ESA, and T_1_ ESA bands are shown in Figure 1(d) and Figure S9 in the SI. Instrument response function (IRF) convoluted exponential fitting yields a time constant for intersystem crossing (τ_ISC_) of 1.5 ± 0.1 ps and a time constant corresponding to internal conversion and vibrational relaxation within the triplet manifold (τ_IC+VR_) of 10.9 ± 0.2 ps. Due to the limited time delay range of our TA setup, we were unable to determine the lifetime of the T_1_ state directly; however, it has previously been reported to be approximately 2.4 μs.^14^

Ground-state bleach (GSB) recovery kinetics can provide critical insight into the photophysical processes of a molecular system. In the case of DDQ, direct monitoring of the GSB via TA spectroscopy is challenging due to spectral overlap with strong ESA bands from both the singlet and triplet states near 375 nm, as well as the relatively low probe intensity below 350 nm in the white-light continuum used. However, we successfully captured GSB features using TRIR spectroscopy. The Fourier transform infrared (FTIR) spectrum of DDQ in MeCN shows multiple bands in the mid-IR region between 1100 and 1800 cm^–1^, as shown in Figure 3(a). DFT/M06–2X/aug-cc-pVTZ/PCM­(MeCN) indicates that the dominant peak in this region is related to the asymmetric C=O stretch of DDQ, while the weak contribution is likely to be connected to the symmetric C=O stretch. In our TRIR experiments, we probed the 1600–1780 cm^–1^ range and observed clear GSB features corresponding to the major band at 1700 cm^–1^ and a secondary band at 1682 cm^–1^. Representative TRIR spectra at selected time delays are shown in Figure 3(b). Notably, no recovery of the GSB is observed within 3.75 ns after photoexcitation. This observation supports the conclusion that rapid ISC effectively prevents repopulation of the ground state (S_0_) via radiative (fluorescence) or nonradiative (IC and VR) decay from the S_1_ state. As a result, the ISC quantum yield (Φ_ISC_) approaches unity.

**Figure 3:**
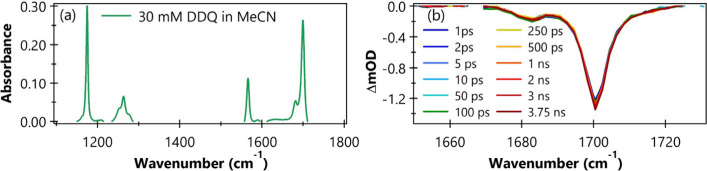
(a) Background-subtracted FTIR spectrum of 30 mM DDQ in MeCN. (b) TRIR spectra of 30 mM DDQ in MeCN following 395 nm photoexcitation, shown at selected time delays.

In summary, we have investigated the early time dynamics of photoexcited DDQ using ultrafast spectroscopic techniques, complemented by extensive quantum chemical calculations. Upon photoexcitation to a ππ* state (S_3_ at the Franck–Condon point), DDQ undergoes rapid diabatic relaxation to the S_1_ (ππ*) state, followed by efficient ISC to the ^3^ *n*_–_π* state with a near-unity quantum yield and an experimentally determined time constant of 1.5 ± 0.1 ps. This ISC is followed by IC to the vibrationally hot T_1_ (ππ*) state, a process that occurs too rapidly to be resolved experimentally. However, vibrational cooling of the T_1_ (ππ*) state has been observed, with a time constant of 10.9 ± 0.2 ps. The resulting relaxed T_1_ (ππ*) state is long-lived and serves as the active photoredox catalyst in the presence of a suitable substrate. The exceptional combination of near-unity Φ_ISC_, high triplet-state reduction potential, and long T_1_ lifetime establishes DDQ as a highly efficient photoredox catalyst and a compelling metal-free alternative to conventional Ir and Ru complexes.

## Supplementary Material



## Data Availability

Data are available at the University of Bristol data repository, data.bris, at 10.5523/bris.2znmhydoqldli27zps0muchl1l.

## Notes

Computational and experimental methods, additional computational results, NMR spectrum of DDQ, decomposition of TA spectra, additional TA spectra and dynamics (PDF)
