# Supplementary material for: Ultrafast Excited-State Dynamics of the Organic Photoredox Catalyst DDQ
Source: J Phys Chem Lett. 2025 Sep 9;16(37):9748–52. doi: 10.1021/acs.jpclett.5c02391 (PMC12451735; doi:10.1021/acs.jpclett.5c02391)
Supplement: Supplementary file 1 [file jz5c02391_si_001.pdf]

Supporting Information for Publication

Ultrafast Excited-State Dynamics of the Organic Photoredox Catalyst DDQ

Deborin Ghosh,* Vera Brieskorn, Charlotte A. Smith, Hallam J. M. Greene, Ria G. Binyahan, Federico J. Hernández, Alastair J. J. Lennox, Basile F. E. Curchod,* Andrew J. Orr-Ewing*

School of Chemistry, University of Bristol, Cantock's Close, Bristol BS8 1TS, UK.

* Authors for correspondence: deborin.ghosh@bristol.ac.uk, basile.curchod@bristol.ac.uk, a.orr-ewing@bristol.ac.uk

Contents **Page**

Supporting Figures

Figure S1 S1
Figure S2 S5
Figure S3 S6
Figure S4 S7
Figure S5 S8
Figure S6 S9
Figure S7 S10
Figure S8 S10
Figure S9 S11

Supporting Sections

Section S1: Computational details S2
Section S2: Experimental Methodology S9

References S11

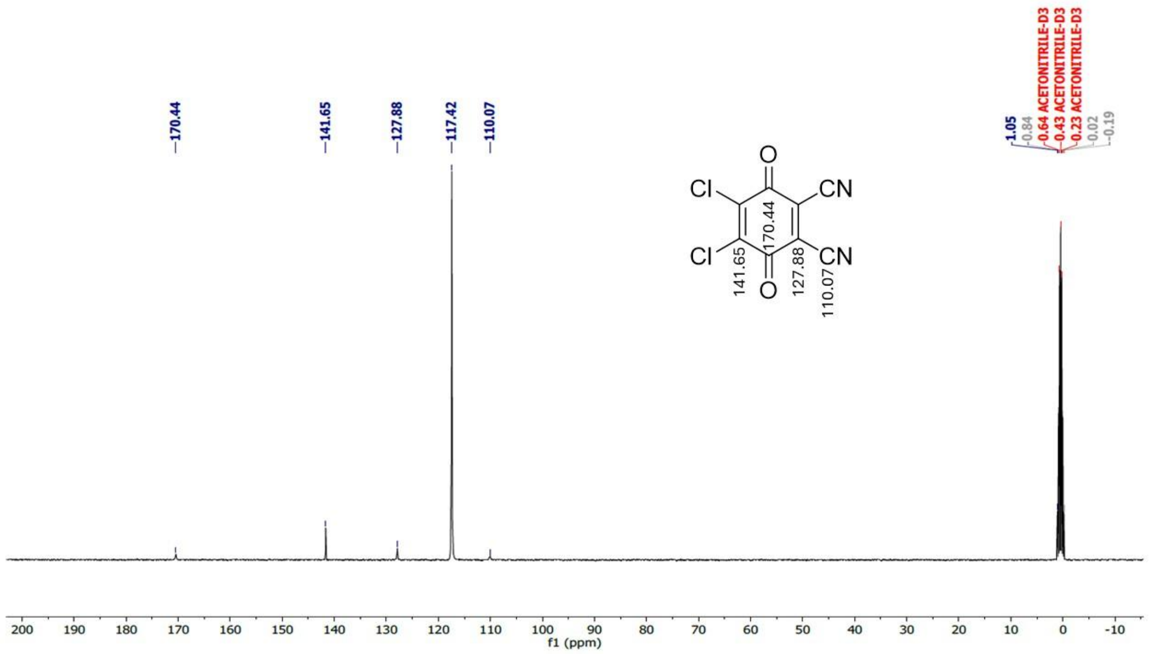

Figure S1: ^{13}C NMR spectrum of 30 mM DDQ in MeCN-d_3. Peak assignments are shown within the figure alongside the chemical structure of DDQ. Signals at δ 117.42 and δ 1.05–0.19 originate from the MeCN-d_3 solvent. The absence of any additional peaks confirms the high purity of the DDQ used in our experiments.

Section S1: Computational details

S1.1: Geometry optimizations and electronic energies

All DFT and LR-TDDFT calculations (unless otherwise specified) were performed with Orca version 5.0.4[1,2] with the default settings of Orca and the default grid. All DFT calculations and LR-TDDFT energy calculations used the ωB97X[3] functional with D4 dispersion correction,[4] and the ZORA Hamiltonian combined with the ZORA-def2-TZVP[5,6] basis set to account for scalar relativistic effects (unless stated otherwise). The Tamm-Dancoff approximation (TDA)[7,8] was used for all LR-TDDFT calculations, and the lowest five singlet excited states were considered. Geometry optimization with LR-TDDFT/TDA employed LibXC with *hyb_gga_xc_wb97x_v*.[9] Calculations including triplet states focused on the five lowest states with this spin multiplicity. The CPCM implicit solvation model[10] was used with the default parameters for MeCN. The $^3n\pi^*$ optimisation was carried out in Orca 6.0 to make use of the *followiroot* option for triplet states, which is not included in the Orca 5.0.4 version. Very tight optimization criteria were required to locate this minimum while avoiding the presence of an imaginary frequency.

To calculate spin-orbit coupling (SOC) matrix elements, a spin-orbit mean-field operator was employed, explicitly including one-electron terms and Coulomb terms using the resolution of identity approximation. Exchange terms were included using one-centre exact integrals that account for the spin-other orbit terms. DFT local correlation terms were not included. As recommended in the literature,[11] the described SOC operator was specified in Orca as 1,3,3,0, which corresponds to the RI-SOMF(1X) operator.

All gas-phase CC2 calculations were perfomed with Turbomole[12] version 7.4.1 with the default convergence criteria and used the SCS approach with the default parameters[13] and the resolution of identity approximation.[14] SCS-CC2 excited state energies and oscillator strengths were obtained with the def2-TZVP basis and auxiliary basis[15], while gas-phase optimisations and frequency calculations were carried out within the def2-SVP[5] basis to reduce the computational cost. SCS-CC2 calculations using the COSMO implicit solvation model were carried out in Turbomole 7.8 (also with default parameters for the SCS and using the resolution of the identity). An ε value of 36.6 was used for MeCN, matching the value used in Orca 5.0.4. The default refractive index and atom type radii were used within the post-SCF scheme. The excited-state absorption from the $T_1(^3\pi\pi^*)$ geometry was calculated with SCS-CC2/def2-TZVP/COSMO[16] using the same settings as specified above.

All optimized geometries (obtained with DFT, LR-TDDFT, or SCS-CC2) were confirmed to be local minima by performing frequency calculations and obtaining only real frequencies. Assignments of IR transitions for the ground-electronic state used a minimum-energy geometry optimized with DFT/M06-2X/aug-cc-pVTZ[17,18]/PCM(MeCN) with the DefGrid3 option and frequencies calculated at the same level of theory (scaled by a factor of 0.9557).

All natural transition orbitals (NTOs) from Turbomole or Orca calculations were obtained with TheoDORE[19] and visualized using VMD.[20]

We note that for the discussion in the main text, we often use a diabatic picture, i.e., one based on the character of the electronic states considered. When further clarity is needed, we include

information about the adiabatic state and the geometry discussed. For example, S_1(^{1}nπ*) means the S_1 optimized geometry exhibiting nπ* character.

S1.2: Absorption spectrum

The absorption spectrum presented in Figure 1(a) (main text) was obtained using the nuclear ensemble approach (NEA).[21] A harmonic Wigner distribution was constructed from the optimized ground-state geometry (DFT/ωB97X-D4/def2-TZVP/PCM(MeCN)) and corresponding vibrational frequencies using the in-house code Harmonwig [https://github.com/ispg-group/harmonwig/]. 1000 nuclear geometries were sampled from this distribution. Excitation energies and transition moments were calculated for each of these 1000 sampled structures with LR-TDDFT/TDA/ωB97X-D4/def2-TZVP/PCM(MeCN), considering the five lowest singlet excited electronic states (according to the equation found in previous literature,[22] only the lowest three were subsequently used for the calculation of the absorption spectrum). Each transition was broadened with a Gaussian function characterized by a width of 0.05 eV. We stress here that a spectrum calculated within the NEA lacks any vibronic structure, as the method does not incorporate overlaps between vibrational wavefunctions. Instead, the features observed in a spectrum obtained with the NEA are the results of the limited number of geometries sampled from the corresponding ground-state distribution.

S1.3: Search for a minimum-energy structure in S_1 with nπ* character

While the lowest-energy geometry for the first excited state (S_1) has ππ* character, all our attempts to try to locate a minimum on S_1 with nπ* character using an implicit solvent for acetonitrile were unsuccessful – e.g., S_1 optimization initiated from the gas-phase S_1(nπ*) or S_0 geometries, optimization started from a higher singlet state with nπ* character, S_1 optimization beginning from the geometry of the ^{3}nπ* minimum. These various failures in locating a minimum on S_1 with nπ* character indicate that such a minimum may not exist within the LR-TDDFT/PCM approach deployed here.

S1.4: Interpolated pathways

All interpolated pathways were obtained using the geodesic interpolation approach introduced in Reference [23]. Interpolations were performed between the critical structures (minimum-energy geometries for various electronic states, namely FC, ^{1}nπ*, ^{3}ππ*, ^{3}nπ* and, for some pathways discussed in the following, another alternative ^{1}nπ*' state), obtained with LR-TDDFT in implicit solvation. Additional pathways were calculated between critical geometries obtained with SCS- CC2 in the gas phase (in the order ^{1}nπ*', FC, ^{1}nπ*, ^{3}ππ*, and ^{3}nπ*). In every interpolation, 15 interpolated geometries were produced between each pair of critical geometries of interest.

The interpolated pathway presented in the main text was carried out between the critical structures optimized with LR-TDDFT/TDA using a PCM model for MeCN (the geometries forming this pathway are available as part of the supporting material of the article). Electronic energies were recalculated with SCS-CC2/def2-TZVP/COSMO(MeCN) using the geometries forming the LR-TDDFT pathway. This procedure was adopted to improve the depiction of energy differences between singlet and triplet electronic states, given the notorious variability in accuracy observed with LR-TDDFT/TDA in describing triplet and singlet states.[24]

Throughout the entire interpolated pathway, the maximum value for the D1 diagnostic (CC2) was 0.0875. For the percentage of single excitation character in the response calculation, a minimum

of 88.2% was encountered for the singlet states and 89.5% for the triplets. These values fall within a tolerance range for CC2, building confidence in the choice of this method to describe DDQ.[25, 26]

S1.5: Calculation of intersystem crossing transition rates

The intersystem crossing transition rate constant (k_{ISC}) between the singlet ($^1\pi\pi^*$) and triplet ($^3n\pi^*$) states discussed in the main text was obtained by localizing the minimum for each state with LR-TDDFT/TDA/ωB97X-D4/def2-TZVP/PCM(MeCN), and calculating their respective vibrational frequencies and the spin-orbit coupling matrix element between these two electronic states at the S_1($^1\pi\pi^*$) minimum geometry. This information was then used within FCclasses3,[27] using an in-house interface between FCclasses and Orca, to determine the intersystem crossing transition rate based on a time-dependent formalism and the adiabatic Hessian approximation. A temperature of 298 K was employed, together with Gaussian broadening functions using a broadening factor of 0.01 eV. As a test, we also calculated the intersystem crossing rate for gas-phase DDQ, this time between $^1n\pi^*$ and $^3\pi\pi^*$ states and using SCS-CC2 optimized geometries (using an in-house interface between FCclasses and Turbomole), obtaining a time constant of 3.0 ps (see Figure S5) and validating the fast intersystem crossing process between these two electronic-state characters. LR-TDDFT/TDA/ωB97X-D4/def2-TZVP was employed to determine the magnitude of spin-orbit coupling between these two states (43.1 cm^{-1}) at the S_1($^1n\pi^*$) optimized geometry. We emphasize that the approximations employed for determining the intersystem crossing rate mean that a direct comparison between the calculated value and the experimental time constant should be made with care – the calculations reported here should instead be viewed as a validation of a fast intersystem crossing process.

S1.6: Additional computational information

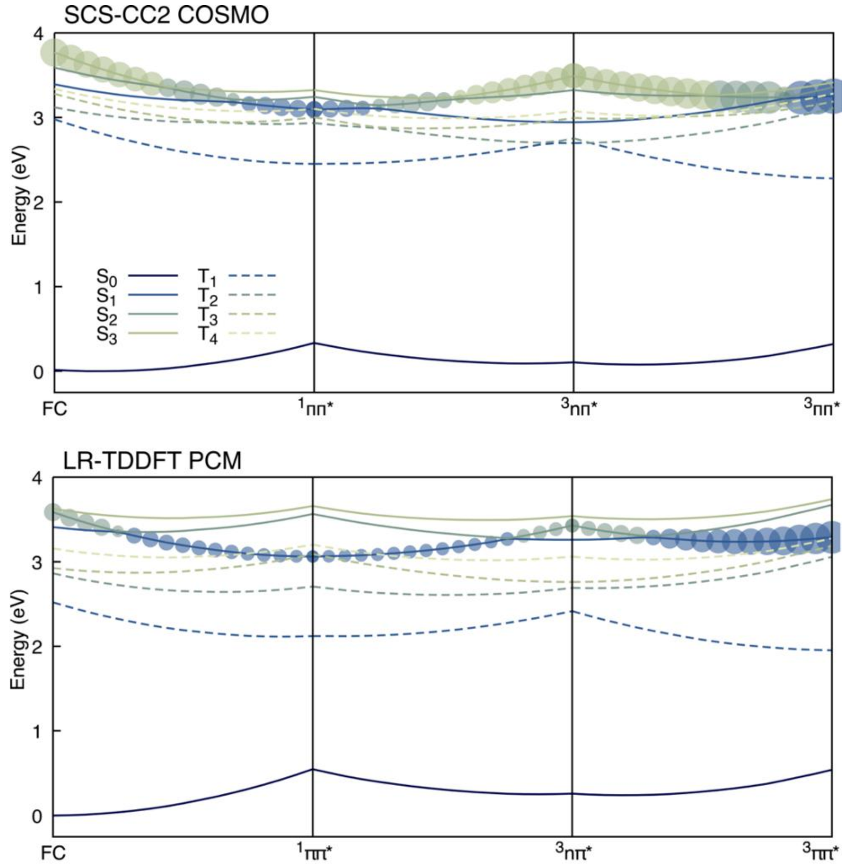

Figure S2: Comparison between interpolated pathways obtained with SCS-CC2/COSMO(MeCN) (upper panel) and LR-TDDFT/PCM(MeCN) (lower panel) electronic energies and oscillator strengths. The geometries forming the interpolated pathways are the same as those used in the main text and are based on critical geometries optimized with (LR-TD)DFT/PCM(MeCN). Circles indicate oscillator strengths, with their radius being proportional to the magnitude of the oscillator strength between the ground electronic state and the excited electronic state considered at a given molecular geometry (a scaling factor of 200 and 400 was used for SCS-CC2 and LR-TDDFT, respectively). Overall, SCS-CC2 and LR-TDDFT yield a similar picture for the potential relaxation pathways – excitation to a ππ* bright state in the FC region, relaxation to the $^1\pi\pi^*$ state where ISC is favoured, and relaxation to the $^3\pi\pi^*$ state – while differences between electronic energies can be observed. The LR-TDDFT singlet/triplet (S/T) gaps (maximum value of 1.79 eV for T_1-S_3 at the ππ* structure) are higher than with SCS-CC2 (energy gap of 0.90 eV between T_1-S_3 on average over the four critical geometries). This tendency resonated with the tendency for LR-TDDFT to underestimate triplet excitation energies.[24]

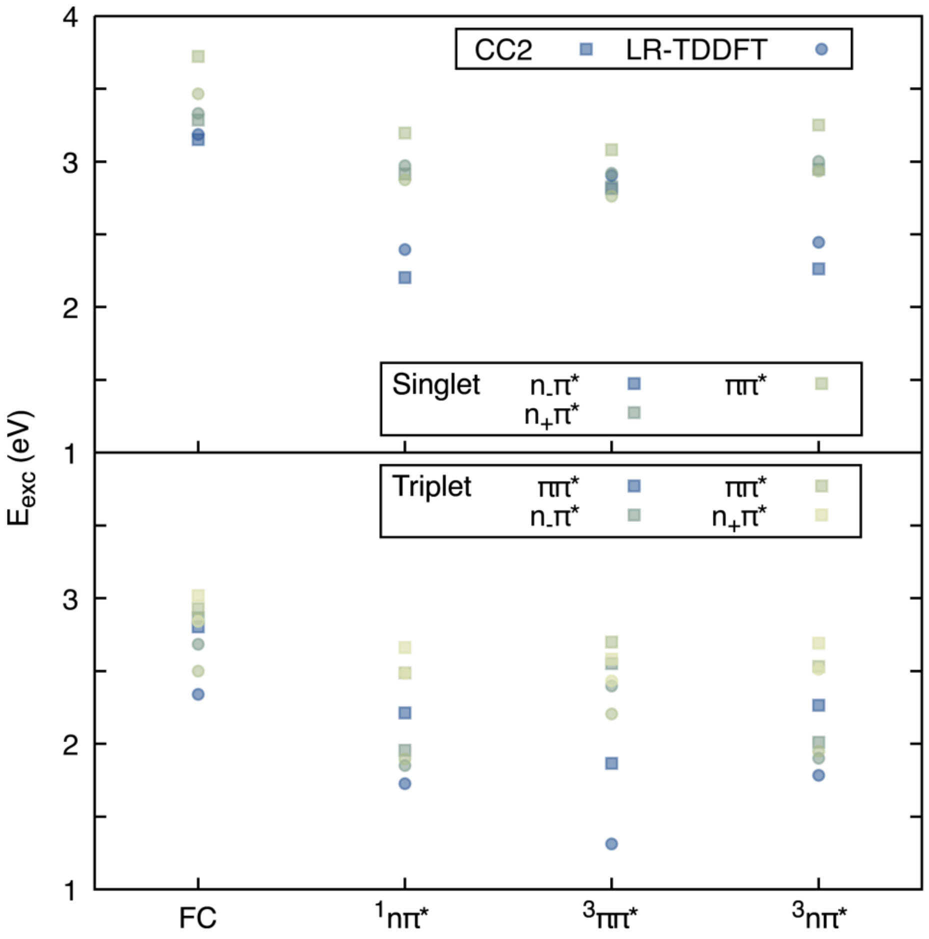

Figure S3: Comparison between the excitation energies obtained with SCS-CC2/def2-TZVP and LR-TDDFT/TDA/ωB97XD4/ZORA/def2-TZVP using the geometries optimized with SCS-CC2 in the gas phase. Singlet $S_{1\text{-}3}$ (upper panel) and triplet $T_{1\text{-}4}$ (lower panel) excitation energies are colour coded according to the character specified in the legend. Squares indicate SCS-CC2 and circles LR-TDDFT excitation energies. The lower panel shows that triplet excitation energies obtained with LR-TDDFT are always lower than the corresponding SCS-CC2 energies; ππ* states appear to be more affected than nπ* states. The comparison proposed here aligns well with the comparison in Figure S2 and indicates that the differences between LR-TDDFT and SCS-CC2 excitation energies are consistent (independently of the geometry optimization method used), giving further confidence in the use of SCS-CC2 on top of LR-TDDFT/PCM(MeCN) optimized structures.

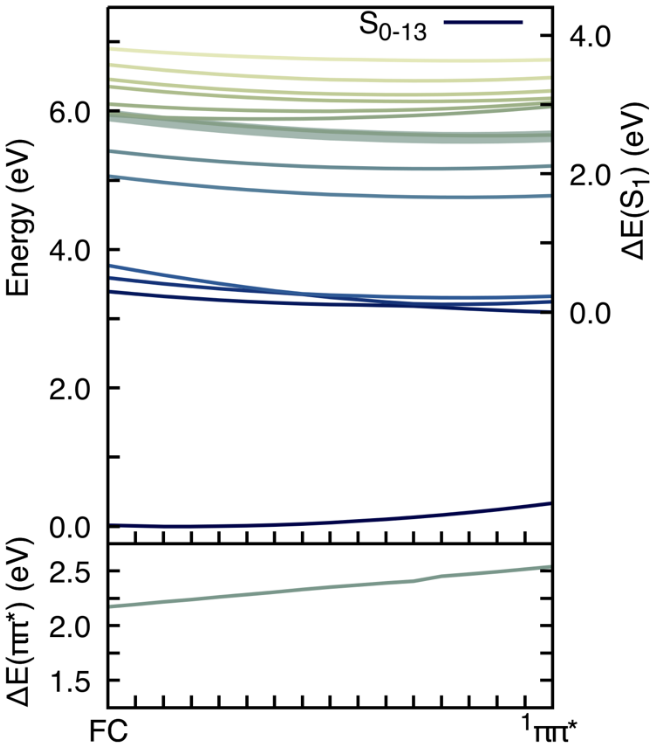

Figure S4: Interpolated pathways obtained with SCS-CC2/COSMO(MeCN) electronic energies and oscillator strengths for the lowest 13 excited electronic states, showing the higher excited electronic states that can be targeted in the excited-state absorption process. The geometries forming the interpolated pathway are the same as those used in the main text and based on critical geometries optimized with (LR-TD)DFT/PCM(MeCN), here the S_0 optimized geometry (FC) and the S_1(ππ*) optimized geometry. The right-hand side ordinate for the upper panel shows the electronic energy difference with respect to the energy of DDQ at its optimized S_1(ππ*) geometry. The lower panel shows the energy difference between the lowest electronic state with a ππ* state along the pathway (S_3 for the FC geometry until S_1 at the optimized ππ* geometry) and the higher energy ππ* state with significant oscillator strength (highlighted with a thicker curve in the upper panel). This excited-state absorption lies in the experimental ESA range and shifts solely by 0.37 eV from 2.17 eV to 2.54 eV along this segment of the interpolated pathway. We note that a shift towards lower wavelengths is also observed for the experimental ESA band assigned to the S_1 state.

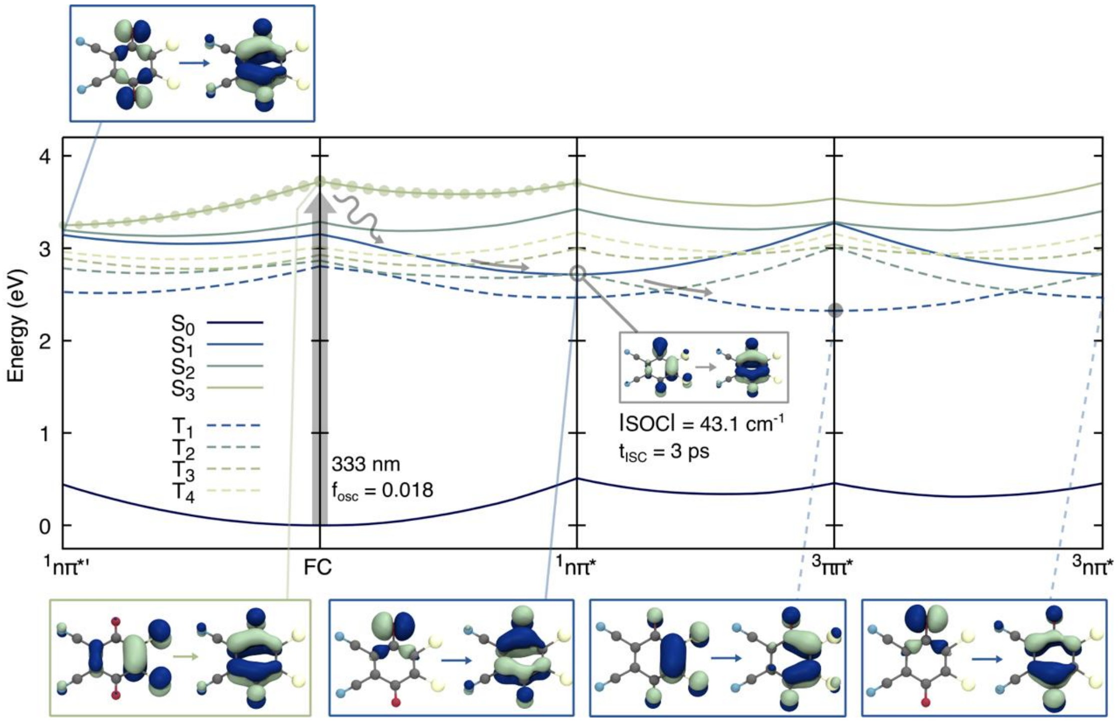

Figure S5: Gas-phase deactivation pathway for DDQ. Interpolated pathway for DDQ in the gas phase based on SCS-CC2 optimized geometries for S_1 (^{1}nπ*'), S_0 (FC), S_1 (^{1}nπ*), T_1 (^{3}ππ*), and T_1 (^{3}nπ*) and using SCS-CC2 electronic energies and oscillator strengths (circles). This pathway indicates that the nonradiative decay of DDQ in the gas phase operates with electronic states of similar character to those in solution, but with different energy ordering. The S_1 minimum exhibits ^{1}nπ* character and is connected to a ^{3}ππ* state via efficient intersystem crossing. Arrows indicate the suggested deactivation pathway from S_3 (FC) to T_1 (^{3}ππ*). The bottom inset shows the natural transition orbitals (NTOs) for the electronic state of interest (see colour code) for each optimized structure. No minimum on S_1 with ^{1}ππ* character could be located in the gas phase with SCS-CC2. This interpolated path based on SCS-CC2 structures suggests that a relaxation pathway involving efficient ISC is equally possible in the gas phase with an ISC time constant of 3 ps. This pathway also indicates that if a minimum in S_1 with an nπ* character were to exist in solution, fast intersystem crossing would still be possible towards the triplet manifold.

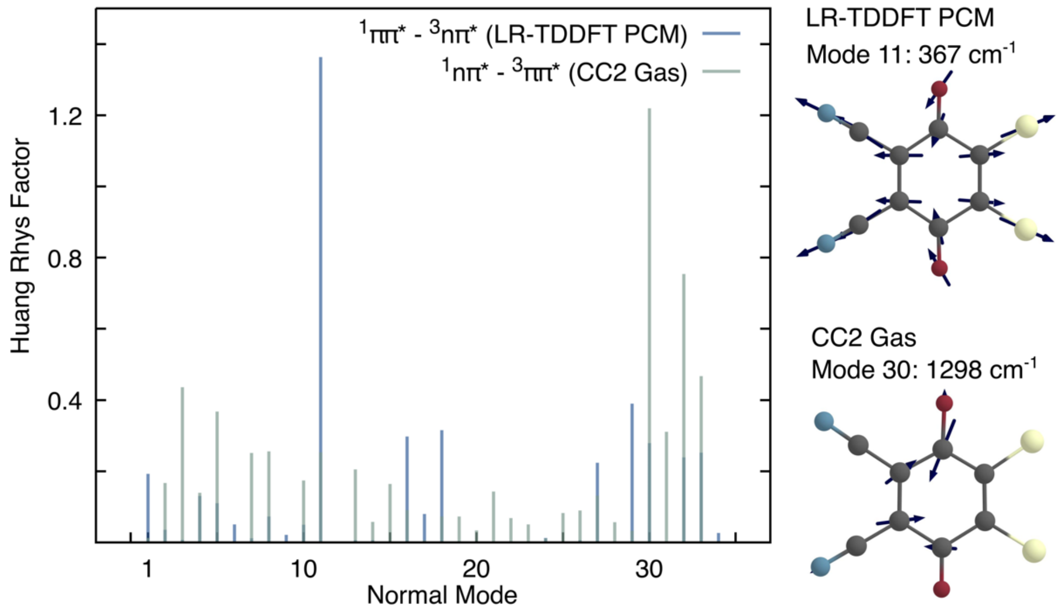

Figure S6: Huang-Rhys (HR) factors for the ISC process obtained from LR-TDDFT/PCM(MeCN) (from the $^1\pi\pi^*$ to the $^3n\pi^*$ minimum, as discussed in the main text) and for the ISC process discussed in Figure S5 above based on SCS-CC2 calculations in the gas phase (from the $^1n\pi^*$ to the $^3\pi\pi^*$ minimum). A high HR factor indicates a strong contribution of the corresponding normal mode to the nonradiative transition. The right-hand side inset displays the normal mode with the highest HR factor for LR-TDDFT/PCM(MeCN) and SCS-CC2. This analysis suggests that no out-of-plane motion is involved in the ISC process described at these levels of theory, which provides insight into why the ISC process can be as fast as calculated (and observed experimentally).

Section S2: Experimental Methodology

Transient electronic absorption (TA) and time-resolved mid-infrared absorption (TRIR) spectra were recorded at the University of Bristol using an ultrafast laser setup. A detailed description of the system is provided in the Supporting Information of reference.[28]

Briefly, femtosecond laser pulses were generated by a Coherent Astrella regenerative amplifier system (1 kHz, 800 nm, 7 W, 35 fs pulse width), seeded by a Ti:sapphire oscillator. The pump beam at 395 nm was produced via an optical parametric amplifier (OPA, Coherent OPerA Solo), delivering approximately 200 nJ of energy at the sample. White-light continuum (WLC) probe pulses, covering a spectral range of 350–740 nm for TA measurements, were generated by focusing a portion of the 800 nm beam into a calcium fluoride (CaF_2) plate. A tunable broadband mid-IR output generated from a second OPA was used as the probe for TRIR measurements.

The pump and probe beams were spatially and temporally overlapped at the sample position. The transmitted probe was collected either by an Andor Shamrock 163 spectrometer equipped with a 1024-element photodiode array (Entwicklungsbüro Stresing) for TA spectra, or by a 128-element mercury cadmium telluride (MCT) detector (Infrared Systems Development Corporation) for TRIR spectra. The relative polarization between the pump and probe beams was set to the magic angle (54.7°) to eliminate rotational contributions. The instrument response function (IRF) was determined to be approximately 120 fs by fitting solvent-only measurements. IRF-convoluted exponential fitting functions were used to extract the time constants associated with various excited-state dynamic processes. This setup enabled time-resolved measurements over a window spanning 100 fs to 3.75 ns, with time delays controlled via an optical delay stage.

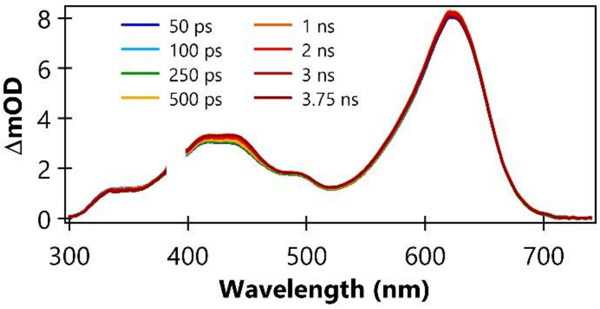

Figure S7: TA spectra of 30 mM DDQ in MeCN following 395 nm excitation, recorded at later time delays (50 ps to 3.75 ns).

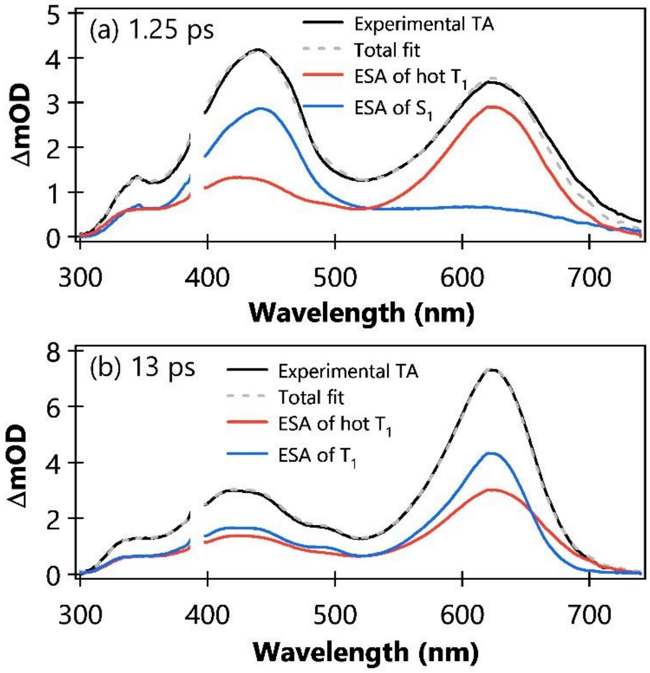

Figure S8: Spectral decomposition of TA features for 30 mM DDQ in MeCN following 395 nm photoexcitation, shown at time delays of (a) 1.25 ps and (b)13 ps. The inset keys identify the experimental spectra, the basis functions used and the resulting fits. The spectrum is masked around 395 nm where pump laser scatter contributes.

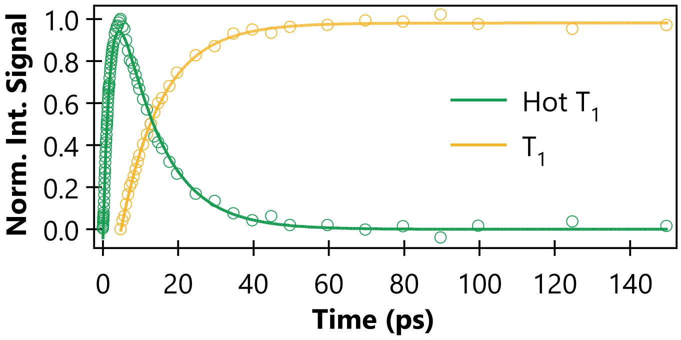

Figure S9: Kinetics of vibrationally hot T_1 and minimum energy T_1 state populations, derived from time-dependent integrated and normalized band intensities (○) following spectral decomposition using KOALA software.[29] Solid lines represent bi-exponential (hot T_1) and mono-exponential (T_1) fits.

References

(1) Riley, K. E.; Hobza, P. Noncovalent interactions in biochemistry. *WIREs Comput. Mol. Sci.* **2011**, *1* (1), 3-17. DOI: 10.1002/wcms.8.

(2) Neese, F. Software update: The ORCA program system—Version 5.0. *WIREs Comput. Mol. Sci.* **2022**, *12* (5), e1606. DOI: 10.1002/wcms.1606.

(3) Mardirossian, N.; Head-Gordon, M. ωB97X-V: A 10-parameter, range-separated hybrid, generalized gradient approximation density functional with nonlocal correlation, designed by a survival-of-the-fittest strategy. *Phys. Chem. Chem. Phys.* **2014**, *16* (21), 9904-9924. DOI: 10.1039/C3CP54374A.

(4) Caldeweyher, E.; Ehlert, S.; Hansen, A.; Neugebauer, H.; Spicher, S.; Bannwarth, C.; Grimme, S. A generally applicable atomic-charge dependent London dispersion correction. *J. Chem. Phys.* **2019**, *150* (15), 154122. DOI: 10.1063/1.5090222.

(5) Weigend, F.; Ahlrichs, R. Balanced basis sets of split valence, triple zeta valence and quadruple zeta valence quality for H to Rn: Design and assessment of accuracy. *Phys. Chem. Chem. Phys.* **2005**, *7* (18), 3297-3305. DOI: 10.1039/B508541A.

(6) Pantazis, D. A.; Chen, X.-Y.; Landis, C. R.; Neese, F. All-Electron Scalar Relativistic Basis Sets for Third-Row Transition Metal Atoms. *J. Chem. Theory Comput.* **2008**, *4* (6), 908-919. DOI: 10.1021/ct800047t.

(7) Hirata, S.; Head-Gordon, M. Time-dependent density functional theory within the Tamm–Dancoff approximation. *Chem. Phys. Lett.* **1999**, *314* (3), 291-299. DOI: 10.1016/S0009-2614(99)01149-5.

(8) Petrenko, T.; Kossmann, S.; Neese, F. Efficient time-dependent density functional theory approximations for hybrid density functionals: Analytical gradients and parallelization. *J. Chem. Phys.* **2011**, *134* (5), 054116. DOI: 10.1063/1.3533441.

(9) Lehtola, S.; Steigemann, C.; Oliveira, M. J. T.; Marques, M. A. L. Recent developments in libxc — A comprehensive library of functionals for density functional theory. *SoftwareX* **2018**, *7*, 1-5. DOI: 10.1016/j.softx.2017.11.002.

(10) Garcia-Ratés, M.; Neese, F. Effect of the Solute Cavity on the Solvation Energy and its Derivatives within the Framework of the Gaussian Charge Scheme. *J. Comput. Chem.* **2020**, *41* (9), 922-939. DOI: 10.1002/jcc.26139.

(11) Neese, F. Efficient and accurate approximations to the molecular spin-orbit coupling operator and their use in molecular g-tensor calculations. *J. Chem. Phys.* **2005**, *122* (3), 034107. DOI: 10.1063/1.1829047.
(12) Balasubramani, S. G.; Chen, G. P.; Coriani, S.; Diedenhofen, M.; Frank, M. S.; Franzke, Y. J.; Furche, F.; Grotjahn, R.; Harding, M. E.; Hättig, C.; et al. TURBOMOLE: Modular program suite for ab initio quantum-chemical and condensed-matter simulations. *J. Chem. Phys.* **2020**, *152* (18), 184107. DOI: 10.1063/5.0004635.
(13) Hellweg, A.; Grün, S. A.; Hättig, C. Benchmarking the performance of spin-component scaled CC2 in ground and electronically excited states. *Phys. Chem. Chem. Phys.* **2008**, *10* (28), 4119-4127. DOI: 10.1039/B803727B.
(14) Hättig, C.; Weigend, F. CC2 excitation energy calculations on large molecules using the resolution of the identity approximation. *J. Chem. Phys.* **2000**, *113* (13), 5154-5161. DOI: 10.1063/1.1290013.
(15) Weigend, F.; Häser, M.; Patzelt, H.; Ahlrichs, R. RI-MP2: optimized auxiliary basis sets and demonstration of efficiency. *Chem. Phys. Lett.* **1998**, *294* (1), 143-152. DOI: 10.1016/S0009-2614(98)00862-8.
(16) Khani, S. K.; Faber, R.; Santoro, F.; Hättig, C.; Coriani, S. UV Absorption and Magnetic Circular Dichroism Spectra of Purine, Adenine, and Guanine: A Coupled Cluster Study in Vacuo and in Aqueous Solution. *J. Chem. Theory Comput.* **2019**, *15* (2), 1242-1254. DOI: 10.1021/acs.jctc.8b00930.
(17) Kendall, R. A.; Dunning, T. H., Jr.; Harrison, R. J. Electron affinities of the first-row atoms revisited. Systematic basis sets and wave functions. *J. Chem. Phys.* **1992**, *96* (9), 6796-6806. DOI: 10.1063/1.462569.
(18) Woon, D. E.; Dunning, T. H., Jr. Gaussian basis sets for use in correlated molecular calculations. III. The atoms aluminum through argon. *J. Chem. Phys.* **1993**, *98* (2), 1358-1371. DOI: 10.1063/1.464303.
(19) Plasser, F. TheoDORE: A toolbox for a detailed and automated analysis of electronic excited state computations. *J. Chem. Phys.* **2020**, *152* (8), 084108. DOI: 10.1063/1.5143076.
(20) Humphrey, W.; Dalke, A.; Schulten, K. VMD: Visual molecular dynamics. *J. Mol. Graph.* **1996**, *14* (1), 33-38. DOI: 10.1016/0263-7855(96)00018-5.
(21) Crespo-Otero, R.; Barbatti, M. Spectrum simulation and decomposition with nuclear ensemble: formal derivation and application to benzene, furan and 2-phenylfuran. *Theor. Chem. Acc.* **2012**, *131* (6), 1237. DOI: 10.1007/s00214-012-1237-4.
(22) Hollas, D.; Curchod, B. F. E. AtmoSpec–A Tool to Calculate Photoabsorption Cross-Sections for Atmospheric Volatile Organic Compounds. *J. Phys. Chem. A* **2024**, *128* (39), 8580-8590. DOI: 10.1021/acs.jpca.4c05174.
(23) Zhu, X.; Thompson, K. C.; Martínez, T. J. Geodesic interpolation for reaction pathways. *J. Chem. Phys.* **2019**, *150* (16), 164103. DOI: 10.1063/1.5090303.
(24) Peach, M. J. G.; Williamson, M. J.; Tozer, D. J. Influence of Triplet Instabilities in TDDFT. *J. Chem. Theory Comput.* **2011**, *7* (11), 3578-3585. DOI: 10.1021/ct200651r.
(25) Köhn, A.; Hättig, C. Analytic gradients for excited states in the coupled-cluster model CC2 employing the resolution-of-the-identity approximation. *J. Chem. Phys.* **2003**, *119* (10), 5021-5036. DOI: 10.1063/1.1597635.
(26) Tuna, D.; Lefrancois, D.; Wolański, Ł.; Gozem, S.; Schapiro, I.; Andruniów, T.; Dreuw, A.; Olivucci, M. Assessment of Approximate Coupled-Cluster and Algebraic-Diagrammatic-Construction Methods for Ground- and Excited-State Reaction Paths and the Conical-Intersection Seam of a Retinal-Chromophore Model. *J. Chem. Theory Comput.* **2015**, *11* (12), 5758-5781. DOI: 10.1021/acs.jctc.5b00022.
(27) Cerezo, J.; Santoro, F. FCclasses3: Vibrationally-resolved spectra simulated at the edge of the harmonic approximation. *J. Comput. Chem.* **2023**, *44* (4), 626-643. DOI: 10.1002/jcc.27027.

(28) Greene, H. J. M.; Ghosh, D.; Sazanovich, I. V.; Phelps, R.; Curchod, B. F. E.; Orr-Ewing, A. J. Competing Nonadiabatic Relaxation Pathways for Near-UV Excited ortho-Nitrophenol in Aqueous Solution. *J. Phys. Chem. Lett.* **2024**, *15* (36), 9153-9159. DOI: 10.1021/acs.jpclett.4c02154.
(29) Grubb, M. P.; Orr-Ewing, A. J.; Ashfold, M. N. R. KOALA: A program for the processing and decomposition of transient spectra. *Rev. Sci. Instrum.* **2014**, *85* (6), 064104. DOI: 10.1063/1.4884516.
